# Use of Real-World Evidence for Drug Regulatory Decisions in China: Current Status and Future Directions

**DOI:** 10.1007/s43441-023-00555-9

**Published:** 2023-08-25

**Authors:** Pei Li, Su Wang, Yuwen Chen

**Affiliations:** 1 School of Business Administration, Shenyang Pharmaceutical University, Shenyang, 110016 People’s Republic of China; 2 Drug Regulatory Research Base of NMPA - Research Institute of Drug Regulatory Science, Shenyang Pharmaceutical University, Shenyang, 110016 People’s Republic of China

**Keywords:** Real-world data (RWD), Real-world evidence (RWE), China’s regulatory decision-making, Methods and guidelines, Opportunities and challenges

## Abstract

Real-world data (RWD) and real-world evidence (RWE) have garnered great interest for supporting drug research and development (R&D) by medical researchers and regulators in recent years. The application and development of RWD/E in drug regulatory decision-making have been vigorously promoted in China. This study seeks to provide a broad overview of how RWE has been contributing to drug regulatory decisions in China. In this paper, we review the development of RWD and RWE, summarize key elements that promote application of RWE, introduce relevant methods and guidelines, elaborate on the opportunities and challenges of RWE in regulatory decision-making in China, and put forward suggestions to promote the application of RWE in China’s regulatory decision-making and to further facilitate innovative drug evaluation and regulation.

## Introduction

Randomized controlled trials (RCTs) are considered as the gold standard for evaluation of drugs. With the advancement of drug development concepts and technologies, limitations of RCTs have become increasingly recognized; for example, the sample size is relatively small, the research environment is idealized, and the cost is relatively high [1]. Real-world data (RWD) and real-world evidence (RWE) come from clinical/medical environments, with a wide range of data sources and a large sample size, which can serve as an important supplement to RCTs. How to use RWE in drug research and development (R&D) and regulatory decision-making has become a growing focus of regulatory authorities, industry, and academia.

The USA was one of the early countries to use RWD/E in regulatory decisions for drugs. The U.S. Food and Drug Administration (FDA) launched the Sentinel Initiative to create a national electronic system for medical product safety surveillance in 2008, in response to a Congressional mandate in the FDA Amendments Act of 2007 [2]. 21st-Century Cures, issued in 2016, requires an evaluation of the potential for use of RWE in regulatory decision-making [3]. Since then, the FDA has launched a series regulator-supported initiatives that drive forward the application of RWE, such as a series of guidelines have been issued to guide and regulate the use of RWE in R&D. Subsequently, the proportion of approvals incorporating RWE have been increasing each year. Studies show that there have been 116 approvals incorporating RWE from January 2019 to June 2021, with 65 of the studies influencing FDA’s final decision and 38 being included in the product label [4].

The GetReal Initiative, in which the European Medicines Agency (EMA) participated in 2013, aims to develop new ways of collecting and synthesizing RWE for earlier use in drug development and healthcare decision-making [5]. EMA launched an adaptive licensing pilot project in 2014, exploring the use of RWD for regulatory decision-making [6]. In 2017, Heads of Medicines Agencies (HMA) and EMA jointly established a working group on Big Data, aiming to use big data (including RWD) to improve regulatory decision-making and raise standards of evidence [7]. In Regulatory Science Strategy to 2025, EM Aset a goal for high-quality RWD and related guidelines to be issued [8, 9]. The use of RWE in regulatory decision-making has become more extensive, as presented in the initial marketing authorization applications of new medicines centrally evaluated in 2018–2019 by EMA. Nearly, all European Public Assessment Reports included RWE for discovery and life-cycle management and about half included RWE for the full development phase and for supporting regulatory decisions at registration [10].

The systematic use of RWE to support drug regulatory decision-making is still in its infancy in China. Recently, national drug regulatory authorities began applying RWE in the review and approval process. In China, the National Medical Products Administration (NMPA) defines data relating to patient health state and/or diagnosis and health care on a daily basis as RWD, and the appropriate and sufficient analysis of these data regarding usage and potential benefits and risks are referred to as RWE. RWD are routinely collected from a variety of sources, such as hospital information systems (e.g., electronic medical records), death registers, medical claims, patient registries, the China Adverse Drug Reaction (ADR) Sentinel Surveillance Alliance (CASSA), population-based cohort studies of a natural experiment, and specific disease cohort databases, omics databases, and outcome data of patient reports and personal mobile devices. The NMPA explicitly requires that only RWD that are fit for purpose—determined primarily by data relevance and reliability can produce RWE; emphasizing that RWD is not the same as RWE. RWE is used to support drug regulatory decisions in China, including (a) providing evidence in support of the effectiveness or safety for a new product approval; (b) providing evidence in support of labeling changes for an approved product; (c) being used as part of a post-marketing requirement to support a regulatory decision; (d) exploring new approaches for clinical research and development of well-known prescriptions/formulas from experience of the traditional Chinese medicine (TCM) practitioner; and (e) others such as to guide clinical research design and locate target groups [11].

This study seeks to understand the role of RWE in drug decision-making in China. In this study, we systematically review the policies, regulations, and guidelines on RWD/E and summarize the active exploration and practice on the application of RWD/E in the regulation of drugs in China.

## Methods

A mixed approach has been employed in this study: involving documentary analysis of government policies, regulations, guidelines, and official information about RWE, RWD, and Real-World Study (RWS) [12] in China, as well as review of the literature from 2009 to March 1, 2023 of publicly available statistics, government documents, and media sources. Publicly available government documents were mainly identified through official websites: The State Council, The People’s Republic of China (http://english.www.gov.cn/), The National Health Commission of the People’s Republic of China (http://en.nhc.gov.cn/), The National Medical Products Administration (https://www.cde.org.cn/), The Center for Drug Evaluation of NMPA (http://english.nmpa.gov.cn/), Hainan Institute of Real-World Data (https://hnrws.cn/en), and Hainan Medical Products Administration (https://amr.hainan.gov.cn/himpa/). Literature was searched through PubMed, Web of Science, and Chinese National Knowledge Infrastructure (CNKI) using the search terms: (‘real world study’ OR ‘real world evidence’ OR ‘real world data’) AND ‘drug’ AND ‘China MeSH’.

## Results and Discussions

### RWE in China’s Drug Policy Regulation

In China, the concept of RWS was introduced by professor Du Wenmin, where the use of a RWS in the safety evaluation of Traditional Chinese Medicine (TCM) injections was discussed; in particular, the Work Plan for the Safety Reevaluation of TCM Injections issued by the NMPA in January 2009 [12, 13]. Subsequently, academics and associations began to explore real-world applications in medical regulation and published a series of guidelines. On October 16, 2017, Technical Specifications for Real-World Research of Traditional Chinese Medicine was drafted by the China Association of Chinese Medicine and published for comment [14]. On August 3, 2018, the Guideline of Real-World Research was issued by the Wu Jieping Medical Foundation and Chinese Thoracic Oncology Group (CTONG), which helped Chinese researchers to better conduct a RWS and improve the quality of the RWS [15]. The China Association of Traditional Chinese Medicine approved a project for the group standard of “Technical Guidelines for Real-world Research of Chinese Patent Medicine” and published the draft for comments on the Professional Committee of Real-world Research of China Association of Traditional Chinese Medicine in October of 2019 [16]. Five real-world data and research technical specifications were published by the China Real-World Data and Research Alliance with support from the NMPA and Hainan Provincial Food and Drug Administration in 2019 [17]. These efforts have laid the foundation for the widespread use of RWE for drug development research in China.

The application for extending the indication of bevacizumab was approved by the NMPA based on RWD in 2018, which was the first attempt to support drug evaluation and decision-making with RWE [18]. In the same year, the NMPA organized representatives from academia, the pharmaceutical industry, and relevant institutions, to launch the drafting of guidance documents on the use of RWE to support drug development and evaluation. Later, the RWS pilot was carried out in Lecheng, Hainan. The application of RWE is actively promoted by regulatory agencies with regulator-supported initiatives that drive forward the application of RWE mainly from the following aspects, described below.

#### RWE has been Included in the Scientific Action Plan for Drug Regulation

The NMPA incorporates and actively promotes RWE into drug development and regulatory decisions and further promotes RWE-related research based on regulatory science in China. They launched the Scientific Action Plan for Drug Regulation in May 2019; methodological research on the use of RWD for clinical evaluation of medical devices was one of the first nine key research projects [19]. Since then, 12 regulatory scientific research bases have been established, relying on well-known domestic universities and scientific research institutions. For example, Hainan Institute of Real-World Data strives to promote research on RWD standards of drugs and medical devices as well as research on data collection, analysis, and application. “Using RWE to Support the Technical Guidelines for the Registration and Marketing Review of New Drugs” was studied by the Institute of Drug Regulatory Science, Shenyang Pharmaceutical University, with the aim of strengthening drug registration and regulation from design requirements and sources of RWD. The Institute of Drug Regulatory Science, China Pharmaceutical University, is focused on the framework of using RWE in drug regulatory decisions. Application of RWD/E in Drug Regulation has been studied by National Institute of Pharmaceutical and Medical Device Regulatory Science, Peking University [20].

In June 2021, research on evaluation methods of RWD supporting TCM, rare disease drugs, innovation, and clinically urgent medical devices was included in the second batch of key projects: to study standards of RWD collection, quality evaluation, processing, and analysis in line with national requisites and to form new evaluation tools, standards, and methods for RWE supporting regulatory decision-making [21]. The symposium of RWS experts of the Scientific Research Base of Drug Device Regulation of the NMPA was held in Beijing, on January 17th, 2022. The discussion focused on the urgent need to solve the ethics and informed consent review in a RWS [22].

#### RWE Application Pilot Work has been Conducted in Lecheng Pilot Zone

In 2018, China took the lead in the RWS pilot work in Boao Lecheng International Medical Tourism Pilot Zone (Lecheng Pilot Zone), Hainan, which is the only region in China that can use licensed medical devices and drugs that have been approved in other countries but not registered in China [23]. The unique national concession policy for import of clinically urgent medical devices makes Lecheng Pilot Zone an important test site for the NMPA to carry out RWE research and development. In June 2019, a pilot project of clinical RWD development was jointly launched by the NMPA and Hainan Provincial Government in Hainan [24].

##### Selection of Pilot Projects

The RWS project of international innovative medicinal products for registration in China was developed in Boao Lecheng. By July 2022, a total of 24 licensed imported drugs and medical devices had been included in the RWD application pilot of Lecheng Pilot Zone. Among them, 8 products have since been approved for market, including three drugs (Pralsetinib capsules, approved in March of 2021; Fluocinolone Acetonide Intravitreal Implants, approved in June of 2022; and Trilaciclib, approved in July of 2022) [25–28].

##### Working Procedures and Mechanisms

In 2021, the Guideline for Conducting Real-world Drug Research Pilot Service in Hainan Boao Lecheng International Medical Tourism Pilot Zone was issued to clarify registration procedures of a RWS for drugs [29]. In March of 2022, the Implementation Plan for the Pilot Work of Clinical Real-world Data Application was jointly issued by the NMPA and Hainan Provincial Government [30], aiming to further push forward the use of RWS drug applications.

##### Infrastructure and Assurance

A series of major research institutions have been established to jointly promote research on RWD/E; for example, the Hainan Institute of Real-World Data, Scientific Research Base of Drug and Medical Device Regulation of the NMPA, Hainan Key Laboratory of Real-World Data Research and Evaluation, Boao Lecheng Real-World Data Research and Innovation Center, and Boao Research Hospital, among others.

##### Workshops and Projects

At the same time, a series of workshops and projects were conducted around the use of RWD/E in drugs and medical devices. For example, the 3rd National Congress on Real-World Data and Studies was held in 2020, with the theme of “Real-World Evidence Supporting Drug Device Regulatory Decision-making—Practice in China” [31]. “Communication Meeting of RWD Application in Drugs” and “Communication Meeting on Real-World Drug Research Varieties” were held in 2021[32]. These Conferences discussed the challenges and countermeasures in a RWS of medicinal products and further assistance in product registration. The Boao International Pharmaceutical Devices Real-World Research Conference, which focused on the theme of “Real-World Data Research and Innovative Development of drug device regulation,” was held in 2022 [33].

##### Talent Cultivation and Training

Hainan Institute of Real-World Data has cooperated with Hainan University to set up the first postgraduate training center for “RWD application” in China. In 2021, the joint training target of 10 master’s and 5 doctoral students was achieved [34]. Aiming to help inform the design and implementation of a RWS that could be used to support the development of safe and effective drug products in China; a series of trainings about using RWS/D/E in regulatory decisions have been conducted. Mainly sponsored by Hainan Real-World Data Research Institute (since 2021), the trainings cover basic theory, methodology, data quality control, RWD platform operations, and related processes, ethics, and RWS practice, among other things [35–45].

##### Cooperation and Fund Support

A Consortium for RWD Research on infectious diseases, hepatobiliary and pancreatic diseases, ophthalmology, respiratory diseases, and cancer was established in 2021 [46]. By the end of 2021, most (almost 80%) of the world's top 30 pharmaceutical and medical device companies had established close cooperative relationships with Lecheng Pilot Zone [34]. For example, Novartis signed a memorandum of strategic cooperation with the Boao Lecheng Authority to accelerate the introduction of Novartis' innovative products to the Lecheng Pilot Zone platform [47]. At the same time, the eSource Record (ESR) project of Boao Lecheng Clinical Research Center was officially released. By using artificial intelligence technologies such as natural language processing, it is committed to solving data quality throughout the life cycle and integrating multi-source data [48].

#### RWD Research in Greater Bay Area

In 2019, the Guangzhou Municipal Health and Wellness Commission established the Guangzhou Real-World Research Center of Chinese Medicine in the Greater Bay Area, which is the first research institution in China focusing on RWS in Chinese medicine based on family doctors [49]. On August 27, 2021, the “Hong Kong and Macao Drug and Machinery Pass” policy was officially extended to allow drugs that are in urgent clinical need and have been listed in Hong Kong and Macao but are not listed on the mainland to be used in Guangdong Province after approval by the Guangdong Provincial People’s Government [50]. The eligible designated medical institutions in the Greater Bay Area provide advantageous conditions for RWD drug development. On July 22, 2022, the ethics committee of the Real-World Research Center of Chinese Medicine in the Greater Bay Area of Guangzhou was established [51]. 3 days later, the Real-World Research Institute of Pharmaceutical and Medical Devices in the Greater Bay Area of Guangdong, Hong Kong, and Macao was established at Jinan University to further promote RWS [52].

#### Technical Guiding Principles have been Issued Continually

The Guiding Principle for Real-world Evidence to Support Drug Development and Evaluation (Trial) was issued by NMPA in 2020 [11]. Subsequently, a series of guidelines related to RWD/E have been issued to clarify the technical requirements (Table 1) [53–63].

**Table 1:** List of Relevant RWD/RWE/RWS Regulations and Guidelines

No	Issue Date	Technical Guidelines	Related Contents
1	2020.01.07	Technical Guideline for Real-world Evidence to Support Drug Development and Review (Trial)	Relevant definitions of RWS/RWD/RWE; Common sources of RWD; Data Standard; Data Suitability; Uses RWE in drug regulatory decisions; Basic design for RWS; Evaluation of RWE; Communication with regulatory agencies
2	2020.08.31	Technical Guidance for Using RWE to Support R&D and Regulatory Review of Pediatric Drugs (Trial)	The difference and integration of RWS and RCT; Uses of RWS in the development of pediatric drug; Cases; other points that need attention
3	2021.04.15	Technical Guideline for Real-world Data to Generate Real-world Evidence (Trial)	Common sources and application challenges of RWD; Applicability Evaluation of RWD; RWD governance; Compliance, security, and quality management systems for RWD; Communication with regulatory agencies
4	2022.01.04	Guideline for the Application of Patient-reported Outcomes in Drug Clinical Studies (Trial)	This guideline applies to the use of PRO as an endpoint in support of drug clinical research, including clinical trials and RWS, which clarifies the use of PRO/ePRO in RWS
5	2022.01.06	Technical Guideline for Clinical Research and Development of Rare Disease Drugs (Trial)	This guideline involved that in addition to serving as external control data for single-arm trials, RWS can be used to support the addition of rare disease indications for marketed drugs
6	2022.04.29	Technical Guideline for Communication under Registration Review Evidence System Based on ‟Three Combinations” (Trial)	Encouraging the adoption of RWS to capture human experience data and communication with NMPA is involved in this guideline
7	2022.04.29	Clinical Research and Development of New Drugs for Traditional Chinese Medicine Compound Preparations Based on Human Use Experience (Trial)	A new drug R&D strategy of traditional Chinese medicine combines real-world research and randomized clinical trials is proposed. Based on the time of study data acquisition, it classifies studies as based on past clinical studies with empirical data (retrospective or prospective observational studies or retrospective prospective observational studies, RCT, PCT)) and prospective studies (prospective observational studies, RCT, PCT)
8	2022.06.06	Technical Guideline for Statistics of Clinical Research on Drugs for Rare Diseases (Trial)	The statistics consideration of RWS in Clinical Research on Drugs for Rare Diseases
9	2022.06.20	Technical Guideline for the Suitability of Single-arm Clinical Trials to Support the Marketing Application of Antineoplastic Drugs (Draft for Comment)	Considerations for external control using RWE are clarified
10	2022.12.21	Guideline for Natural History Studies in Drug Development for Rare Diseases (Draft for Comment)	The consideration of RWS in Natural History Studies
11	2023.02.16	Guideline for Real-World Research Design and Protocol Framework for Drugs (Trial)	The research design of the main types of real world (observational study design, pragmatic clinical trial, the single-arm study design); The main structure of RWS plan framework (abstract, background, purpose, hypothesis……); Other considerations; The communication with regulators
12	2023.02.16	Technical guideline for communication of real-world evidence support to drug registration applications (Trial)	This guideline applies to the use of RWE as support for validity and/or critical evidence of safety evaluation to support communication during registration application. Communicate the core issues discussed at the meeting; Requirement for meeting materials; Requirements after meeting

#### Clinical Comprehensive Evaluation Management of Drugs

The use of RWE in comprehensive clinical evaluation has been promoted by the Chinese government. The Guideline for the Management of Comprehensive Clinical Evaluation of Pharmaceutical Products (Trial 2021 edition) has been issued by the General Office of the National Health Commission [64]. RWD are encouraged to be used to conduct a comprehensive analysis in the practical application of drugs, along with utilization data resources from existing national, regional, or provincial databases. In addition, a series of technical guidelines for comprehensive clinical evaluation of specific field drugs, such as pediatric, cardiovascular, and antitumor drugs, has been issued. They provide technical guidance and requirements about the use of RWD in comprehensive clinical evaluation, especially in safety and efficacy analysis [65–67]. RWE is used to promote the improvement of drug policies for drug research and development, production, distribution, and use.

### Case Study of RWE Applications in Drug Approval

RWE studies are considered as part of the evidence package for submissions seeking authorization to market new medicinal products, which is summarized in Table 2. These submissions are reviewed and decisions are rendered primarily by the CDE of NMPA, which focuses on drug products.

**Table 2:** Case Study of RWE Applications in Drug Approval

No	Name	Approval Date by NMPA	Indication	Design of RWS	Relate RWD/RWE in Drug Regulatory Decisions
1	Bevacizumab [68–70]	2018.1	The extension of bevacizumab was approved in combination with platinum-based chemotherapy regimens for the first-line treatment of advanced non-squamous non-small cell lung cancer (NS-NSCLC)	1.Type: retrospective observational study Data source: Shandong Cancer Hospital and Institute Study population:1352 Inclusion/exclusion criteria: All patients were diagnosed with advanced NSCLC and were not suitable for surgical resection of cancerous lesions Exposure: chemotherapy, chemotherapy plus bevacizumab, or supportive care Outcome: complete response (CR), partial response (PR), stable disease (SD), or progression of disease (PD) 2. Type: retrospective observational study Data source: Cancer Hospital, Chinese Academy of Medical Sciences Study population:149 Inclusion/exclusion criteria: Eligible patients were required to be newly diagnosed with stage IIIB or IV cancer with histologically or cytologically confirmed NS-NSCLC Exposure: first-line regimen: bevacizumab-containing (B+) and non-bevacizumab (non-B) Primary outcome: progression-free survival (PFS) 3. Type: retrospective observational study Data source: Jiangsu Cancer Hospital Study population:236 Inclusion/exclusion criteria: the patients with malignant pleural effusion (MPE) or brain metastasis (BM) Exposure: receiving Pem-Pt or B + Pem-Pt Outcome: PFS and/or ORR	These three studies retrospectively analyzed patient data from three hospitals, all of which showed that combination of bevacizumab on the basis of platinum-containing double-drug chemotherapy significantly prolonged PFS and OS compared with chemotherapy alone, consistent with global population data, and no new safety concerns were found. Based on the clinical baseline characteristics of patients, the efficacy of different treatment schemes were analyzed in patient subgroups with different classification criteria, which confirmed the effectiveness and safety of bevacizumab combination therapy in a comprehensive and multi-perspective
2	Pralsetinib Capsules [71]	2021.03	It used to treat a certain type of non-small-cell lung cancer (NSCLC) in adults that has spread to other parts of the body	Type: prospective observational study Data source: Lecheng pilot area Study population: 9 The RWS included a total of nine subjects who were more complex, had more comorbidities, and had lower physical status than the baseline of the key enrollment study	The safety profile of Pratinib observed is consistent with that of BLU-667-1101. In terms of efficacy, the product took effect quickly, and 3 out of 6 patients who could be evaluated had objective remission. Phased study data from the Boao Real-World Study further support the use of this product in RET positive relapsed refractory advanced NSCLC. However, considering the short follow-up time, it is recommended to update the study data in a planned way. Requirements after conditional listing approval: Update the real-world study evidence for NSCLC and PTC indications in Boao Chinese patients as supporting materials and submit a complete study summary report for full approval by supplemental request
3	Qingfei Paidu granules [72]	2021.03	It is used to treat the novel coronavirus pneumonia	Type: retrospective observational study Data source: More than 60 medical institutions in 28 provinces (autonomous regions, municipalities directly under the central government) Study population: 3715	Human empirical evidence based on RWS supported NDA approval
4	Xuanfei Baidu Granules [73]	2021.03	It is used to treat the novel coronavirus pneumonia	Type: retrospective observational study Data source: Isolation ward of Tianjin Haihe Hospital Study population: 40 Inclusion criteria: Patients aged 18 to 80 who have been diagnosed with the novel coronavirus variant Omicron Exposure: Feifuedu granules are used to treat confirmed patients Outcome: clinical symptoms; laboratory index (such as WBC, AST, CT, Nucleic acid turning negative and length of hospitalization)	Xuanfei Baidu granules can significantly improve the clinical symptoms and shorten the negative nucleic acid conversion time and hospital stay It was used as part of a post-marketing requirement to support a regulatory decision
5	Pralsetinib Capsules [74]	2022.03	The extension of bevacizumab was approved to treat a certain type of thyroid cancer in adults and children 12 years of age and older that is getting worse or that has spread to other parts of the body	Not found	Requirements after conditional listing approval: Submit the complete summary report of PTC RWS on this product
6	Fluocinolone Acetonide Intravitreal Implants [75, 76]	2022.06	It is suitable for the treatment of chronic non-infectious uveitis involving the posterior segment of the eye	Type: prospective observational study Data source: Lecheng pilot area The interim report of the RWS, which included data from 28 subjects followed up for 3 months	Submission of NDA application based on overseas clinical trial data and clinical RWD of domestic patients in China The RWS results showed that the recurrence rate of uveitis decreased significantly 3 months after OT-401 implantation, with statistical significance. The RWD in Leicheng accelerated the commercialization of the drug and reduced cost significantly
7	Trilaciclib [77, 78]	2022.07	For prophylactic administration in patients with extensive small-cell lung cancer receiving platinum-containing drugs combined with etoposide regimen to reduce the incidence of chemotherapy-induced myelosuppression	type: prospective observational study data source: Hainan General Hospital study population: 30 Inclusion Criteria: Voluntarily participate and sign informed consent; must be at least 18 years when first dose of Trilaciclib, regardless of gender: Patients with extensive small-cell lung cancer confirmed by histology or cytology; Patients suitable for Trilaciclib combined with platinum/etoposide or Trilaciclib combined with topotecan treatment Exclusion Criteria: Patient is currently participating in other Interventional clinical studies; Patients received systemic chemotherapy other than the regimens recommended in inclusion criteria 4 During Trilaciclib treatment exposure: treacilil treatment Primary Outcome: Incidence of severe neutropenia (SN) [Time Frame: during Trilaciclib plus chemotherapy assessed up to 6 months]; Incidence of severe neutropenia (SN)	The NDA application of Treacilil used RWD as auxiliary supporting evidence. Statistics of RWD showed that only one patient developed severe neutropenia (SN), the most typical myelosuppression induced by chemotherapy, in the first treatment cycle after treacilil treatment, with an incidence of 3.3%, and no pyrogenic neutropenia (FN). Treacillin was equally good for myelosuppression in all patients who completed all cycles of chemotherapy. There were no grade 3 or greater adverse events associated with treacilil, serious adverse events, or adverse events resulting in death

### Challenges and Opportunities in the Application of RWE

#### Challenges

##### RWE Sources and Data Quality Need To Be Further Regulated

In China, the diversity of RWD sources, data from different closed systems, and various management mechanisms and data standardization, among other issues, form obstacles to achieve effective data association and analysis. Furthermore, the process of data recording, collection, and storage of RWD lacks strict quality control, and there may be incomplete data or inconsistent data models and description methods, which form obstacles to the effective use of RWD. It is necessary to establish unified data standards and data quality norms and to improve data quality control methods, in order to ensure the authenticity, accuracy, integrity, and reliability of source data.

##### The Transformation and Application of Lecheng’s RWD Research Results to the Whole of China Need To Be Further Studied

For drugs to be studied in RWS pilots, they must meet two preconditions- “clinical urgent need” and “no similar varieties approved for domestic market” in Lecheng City. With the establishment of the cooperative working mechanism set up by the CDE, Hainan Drug Administration and Lecheng Administration, the “state-province-region” tripartite communication channel has been unblocked, and efficient technical guidance has been provided for pilot manufacturers. Relying on research platforms such as Hainan Institute of Real-World Data and Key Laboratory of Real-World Data Research and Evaluation, top experts and multidisciplinary teams have been brought together to promote the implementation of RWD application pilot work in Lecheng. The above prerequisites and advantages of Lecheng RWS pilot work would be difficult to achieve in other regions of China. There is, therefore, still considerable work that must be done in order to achieve more extensive application of RWE and regulatory agencies should lead the way by developing standards and practices for the evaluation of expanded application.

##### Regulatory Transparency and Technical Guidance Could Be Further Strengthened

In the approved applications incorporating RWE (mainly from Lecheng pilot zone), regulatory considerations on RWE are not explained in the application evaluation public reports or other documents. For example, public reports on Pralsetinib Capsules in 2022 just noted that “Requirements after conditional listing approval: Submit the complete summary report of PTC RWS on this product.” Due to the limited level of detail, it is difficult to know how RWD/E is being judged by the regulators.

##### Collaboration of Regulatory Authorities, Industry, Academia, and Medical Institutions Need to be Farther Strengthened

It is a complicated process to go from RWD to RWE; multiple factors need to be considered, including regulatory application scenarios, clinical elements, data relevance and quality, methodological rationality and rigor, and applicability. In particular, there are still many problems to be solved in the promotion of a RWS. It requires the concerted efforts of regulators, academia, industry, clinical research experts, clinical institutions, data companies, etc. For example, technical guidelines for specific diseases, populations, fields, and technical methods need further study. Also, the orderly, reasonable, and compliant open sharing of medical data need to be promoted, and the data security and rights of patients need to be further protected as ethical and patient privacy issues are involved. Regulatory agencies have begun to tackle these issues in recent years; however, collaboration needs to be farther strengthened to aid in acceptance of RWE for regulatory decisions.

#### Opportunities

In China, RWE application has gained more and more recognition in drug development research in recent years. Authorities and sponsors are jointly exploring the use of RWE in drug registration and regulatory decision-making practice, and translating experience into guiding principles, further guiding practice. The continued push for RWE application in drug regulation in China is part of the broader trend, which is both beneficial and necessary. First, with the deep integration of cloud computing, big data, blockchain, fifth-generation mobile communication (5G), artificial intelligence, and other new generation of information technologies within the medical field, it is helpful for data collection and analysis to be complete and accurate. Second, TCM has been used in China for thousands of years for prophylaxis and treatment of various diseases, accumulating rich experience in human use and massive amounts of data. Most of the new TCM drugs already have a long history of human use experience before marketing application. A RWS fits the characteristics of TCM, which emphasizes integrated regulation, respects individual differences, and follows the characteristics of syndrome differentiation for treatment. The application of RWE in the field of TCM has great potential in future. A scientific and feasible clinical research and development path that combines a RWS and a pragmatic clinical trial will be explored for TCM and a basis for regulatory decision-making will be provided. Third, RWD has been used in comprehensive evaluation management of drugs on a national scale, which brings more extensive and standardized application of RWD, and promotes the sharing of RWD platform and standards. Finally, the marketing authorization holder is required to regularly conduct post-marketing evaluation of the safety, effectiveness, and quality control of the drug already on the market. In addition, post-marketing reevaluation conducted by NMPA also needs RWD to make more comprehensive assessments of the effectiveness, safety, use, and economic benefits of drugs in real-world medical practices and to continually adjust decisions based on RWE. In addition, the use of RWE may also reduce the approval timelines from regulatory agencies. For example, experts from CDE have said that Lecheng’s RWD study assisted Pratinib’s launch to increase the confidence of reviewers and researchers, reduce additional analytical work, accelerate the launch, and enable more patients to benefit earlier. In another motivating example, the use of RWE in Fluocinolone Acetonide Intravitreal Implants accelerated the commercialization of the drug by about a year and a half [75].

Given the above background and trends, we expect that better design and analysis methods and technologies can be discovered and applied, and the current limitations and challenges in the application of RWE can lead to future opportunities. For example, artificial intelligence-based natural language processing, deep learning, intelligent perception, image recognition, and other technologies can provide help to standardize the extraction and transformation of unstructured data in the form of text description. It is becoming easier to obtain high-quality and homogeneous research data and solve the problem of data fusion. The international community attaches great importance to the application of these emerging technologies. For example, FDA, in its 2019–2023 five-year strategy, placed one of their priorities on the development and application of new artificial intelligence technologies, such as natural language processing and machine learning in RWS, and point out regulatory considerations in relevant guidelines [79]. It is suggested that relevant guiding principles should be formulated based on the scientific research results of drug supervision, transparency should be improved, so as to promote the RWS of drugs and improve the evidence system. In addition, the access to and evaluation of relevant data sources or databases are important steps in the design of a RWS and in evaluating a study’s feasibility. It is necessary to promote inter-regional and inter-institutional information system connectivity, mutual recognition and sharing, terminology standardization, and integrated data management, thus eliminating data silos. In addition to the RWE application in Lecheng, it is necessary to further strengthen the cooperation between regulatory authorities, industry, academia, and medical institutions to promote wider application of RWE in drug R&D and regulation.

## Conclusions and Future Directions

In this article, we have attempted to provide a broad overview of the many ways that RWE can contribute to drug regulatory decisions in China. RWD/E has evolved and continues to play a role in recent years; healthcare stakeholders have come to accept and realistically expect RWD/E as part of the evidence that guides decision-making in China. The systematic use of RWE to support drug regulatory decision-making may be in its infancy, but it is growing exponentially. Regulatory authorities, partnering with industry, academia and medical institutions, are positively driving forward the application of RWE. The acceleration and transformation of RWS/D/E will facilitate innovative drug evaluation and regulation.

## References

[1] Sherman RE, Anderson SA, Dal Pan GJ (2016). Real-world evidence—what is it and what can it tell us?. N Engl J Med.

[2] U.S. FOOD & DRUG ADMINISTRATION. FDA's Sentinel Initiative. https://www.fda.gov/safety/fdas-sentinel-initiative/fdas-sentinel-initiative-background.

[3] U.S. Government. 21st Century Cures Act. https://www.Congress.gov/114/plaws/publ255/PLAW-114publ255.pdf.

[4] Purpura CA, Garry EM, Honig N (2022). The role of real-world evidence in FDA-approved new drug and biologics license applications. Clin Pharmacol Ther.

[5] Institute GetReal. RWE for Better Health-Care Decision-Making. https://www.getreal-institute.org/.

[6] European Medicines Agency. European Medicines Agency Launches Adaptive Licensing Pilot Project. https://www.ema.europa.eu/en/news/european-medicines-agency-launches-adaptive-licensing-pilot-project.

[7] European Medicines Agency. HMA-EMA Joint Big Data Taskforce Phase II report “Evolving Data-Driven Regulation”. https://www.ema.europa.eu/en/documents/other/hma-ema-jointbig-data-taskforce-phase-ii-report-evolving-data-driven-regulation_en.pdf.

[8] European Medicines Agency. Regulatory Science Strategy to 2025. Available online: https://www.ema.europa.eu/en/documents/regulatory-proceduralguideline/ema-regulatory-science-2025-strategic-reflection_en.pdf.

[9] European Medicines Agency. Guideline on Registry-Based Studies. https://www.ema.europa.eu/en/guideline-registry-based-studies-0.

[10] Eskola SM, Leufkens HGM, Bate A (2022). Use of real-world data and evidence in drug development of medicinal products centrally authorized in Europe in 2018–2019. Clin Pharmacol Ther.

[11] National Medical Products Administration. Guiding Principle for Real-world Evidence to Support Drug Development and Evaluation (Trial). https://www.nmpa.gov.cn/xxgk/ggtg/qtggtg/20200107151901190.html.

[12] Du WM (2009). Safety of traditional Chinese medicine injections. Drug Eval.

[13] Zhong ZH (2009). Real world research on TCM injections. Chin Prescr Drugs.

[14] China Association of Chinese Medicine. Technical Specifications for Real World Research of Traditional Chinese Medicine. http://www.cacm.org.cn/2017/10/16/4811/.

[15] Chinese Thoracic Oncology Group. Guideline of Real World Research (2018 Edition). https://guide.medlive.cn/guideline/16342.

[16] China Association of Traditional Chinese Medicine. Technical Guidelines for Real-world Research of Chinese Patent Medicine. http://www.cacm.org.cn/2021/06/30/13874/.

[17] Sun X, Tan J, Wang W (2019). Developing technical guidance for real-world data and studies to achieve better production and use of real-world evidence in China. Zhong Guo Xun Zheng Yi Xue Za Zhi.

[18] Center for Drug Evaluation, NMPA. Real World Data Research Continues to Heat Up, and 10 Drugs are Included in the Pilot Program. https://www.cde.org.cn/main/news/viewInfoCommon/.

[19] National Medical Products Administration. The NMPA Launched a Scientific Action Plan for Drug Regulation in China. https://www.cde.org.cn/main/news/viewInfoCommon/e3cd052e638876697399de962b7354a4.

[20] Hainan Institute of Real World Data. The Actions to Facilitate the Scientific Development of Drug Regulation. https://hnrws.cn/notice/1012/1106.html.

[21] National Medical Products Administration. The Second Batch of Key Projects of China Drug Regulatory Scientific Action Plan. https://www.nmpa.gov.cn/xxgk/fgwj/gzwj/gzwjyp/20210628172854126.html.

[22] The Symposium of Real World Research Experts of the Scientific Research Base of Drug Device Regulation of the NMPA, Beijing, January 17th, 2022.

[23] Fu Z, Gao GB, Lin YH. Application of Real-World Clinical Data to the Review and Approval of Medical Devices—Practice in Lecheng Pilot Area of Hainan Province. http://www.cnpharm.com/c/2019-08-06/667151.shtml.

[24] Hainan Institute of Real World Data. The First Medical Device Using Real-World Data has been Approved in China. https://hnrws.cn/zhenyanchengguo/1357/1416.html.

[25] Li MY. Real World Study of Lecheng Pilot Zone. Hainan Ribao. https://www.hainan.gov.cn/hainan/sxian/202207/177ff91fbeb04789b938cd4a6d551cf5.shtml.

[26] CDE of NMPA. Pralsetinib Capsules was Approved by NMPA with Conditions. https://www.cde.org.cn/main/news/viewInfoCommon/4eb0fdbc9d880cd14cb9216c748b7a1c.

[27] CDE of NMPA. Fluocinolone Acetonide Intravitreal Implants-Public Announcement of Marketed Drugs. https://www.cde.org.cn/main/xxgk/postmarketpage?acceptidCODE=5e189ea56f9c4d1fefe3d37de7d437f4.

[28] Hainan Institute of Real World Data. Pilot Variety (Trilaciclib) of Lecheng Approved for Market!. https://hnrws.cn/zhenyanchengguo/1357/2500.html.

[29] Hainan Provincial People's Government. Guideline for Conducting Real-World Drug Research Pilot Services in Hainan Boao Lecheng International Medical Tourism Pilot Zone. https://hnrws.cn/uploads/file/20220316/09/c4f708eb0b700278ca42fa7f0fb6e3e6.pdf.

[30] Hainan Provincial People's Government. Notice on Printing and Distributing Three Programs, Including the Implementation Plan of the Clinical Real World Data Application Pilot Work of Boao Lecheng International Medical Tourism Pilot Zone in Hainan. https://hnrws.cn/1004/1014/1337.html.

[31] West China Hospital of Sichuan University, Hainan Provincial Drug Administration, Hainan Boao Lecheng International Medical Tourism Pilot Zone Administration, China Center for Evidence-based Medicine, West China Branch of ISPOR, Real World Data Research Consortium. 3rd National Congress on Real World Data and Studies. https://2020rws.medmeeting.org/cn.

[32] Hainan Institute of Real World Data. 2021 Real World Research Report for Le City. https://hnrws.cn/event/1422/1439.html.

[33] The Boao International Pharmaceutical Devices Real World Research Conference. https://hnrws.cn/event/gongzuodongtai/2265.html.

[34] Hainan Institute of Real World Data. Master and Doctoral Project Introduction of Hainan Institute of Real World Data. https://hnrws.cn/1009/2237.html.

[35] Hainan Institute of Real World Data. Real World Research Experience Sharing Conference. https://hnrws.cn/event/1422/2453.html.

[36] Hainan Institute of Real World Data. Clinical Research Lecheng Closed-Door Seminar. https://www.sohu.com/a/473088041_664741.

[37] Hainan Institute of Real World Data. Free Trade Port Advanced Workshop on Real World Data Application. https://hnrws.cn/event/gongzuodongtai/2265.html.

[38] Hainan Institute of Real World Data. The Real World Research Drug Clinical Research Training Class. https://hnrws.cn/event/gongzuodongtai/2262.html.

[39] Hainan Institute of Real World Data. Real World Drug Clinical Research Training Class. https://hnrws.cn/event/gongzuodongtai/2264.html.

[40] Hainan Institute of Real World Data. Real World Research Theory and Practice Training Class. https://hnrws.cn/event/gongzuodongtai/2263.html.

[41] Hainan Institute of Real World Data. Training Course on Observational Efficacy Comparative Research Methodology. https://hnrws.cn/event/gongzuodongtai/guanyu2022nianguanchaxingliaoxiaobijiaoyanjiufangf.html.

[42] Hainan Institute of Real World Data. Lecheng Regional Ethics Review Committee and Hainan Real World Data Platform Operation Mechanism and Related Process. https://hnrws.cn/event/1422/2308.html.

[43] Hainan Institute of Real World Data. Training Class on Data Quality Control in Real World Research. https://hnrws.cn/event/gongzuodongtai/2161.html.

[44] Hainan Institute of Real World Data. Real World Data Platform and Real World Research Training Class for Drug Registration. https://hnrws.cn/event/gongzuodongtai/2510.html.

[45] Hainan Institute of Real World Data. Boao Lecheng Real World Research Experience Sharing Event. https://hnrws.cn/event/gongzuodongtai/2512.html..

[46] Six Special Diseases Real World Data Research Alliance Landed in Le City. https://hnrws.cn/notice/1012/1107.html.

[47] Novartis. Boao Lecheng and Novartis Cooperate to Upgrade Version 2.0: Explore Real World Data Research System, Accelerate Innovative Research and Talent Training. https://hnrws.cn/notice/1012/1110.html.

[48] Yao C, Xie HJ, Hao HB (2021). Research on integrated solution tools for real-world data collection, governance and management. China Food Drug Admin Mag.

[49] Guangzhou Municipal Health and Wellness Commission.the Guangzhou Real World Research Center of Chinese Medicine in the Greater Bay Area. https://baike.baidu.com/.

[50] The "Hong Kong and Macao Drug and Equipment Connect" has been in Operation for Nearly a Year, and 29 Drugs and Equipment have been Introduced. Southern Urban Daily. http://mpa.gd.gov.cn/xwdt/xwfbpt/mtzx/content/post_3972151.html.

[51] Inaugural Meeting of the Ethics Committee of the Real World Research Center of Chinese Medicine in Guangzhou City, Greater Bay Area, Guangzhou, July 22, 2022.

[52] Real World Institute of Pharmaceutical and Medical Devices in Guangdong, Hong Kong and Macau Bay Area of Jinan University inaugurated. Jinan University News. https://news.jnu.edu.cn/jnyw/yw/2022/07/11/15112250906.html.

[53] CDE of NMPA. Technical Guideline for Real-world Data to Generate Real-world Evidence (Trial). https://www.cde.org.cn/main/news/viewInfoCommon/2a1c437ed54e7b838a7e86f4ac21c539.

[54] CDE of NMPA. Guideline for Real-World Research Design and Protocol Framework for Drugs (Trial). https://www.cde.org.cn/main/news/viewInfoCommon/14aac16a4fc5b5841bc2529988a611cc.

[55] CDE of NMPA. Technical Guideline for Communication of Real-world Evidence Support to Drug Registration Applications (Trial). https://www.cde.org.cn/main/news/viewInfoCommon/8b59a85b13019b5084675edc912004f1.

[56] NMPA. The Technical Guidance for Using RWE to Support R&D and Regulatory Review of Pediatric Drugs (Trial). https://www.cde.org.cn/main/news/viewInfoCommon/6906389100848948deb49a484197902b .

[57] CDE of NMPA. Guideline for the Application of Patient-reported Outcomes in Drug Clinical Studies (Trial). https://www.cde.org.cn/main/news/viewInfoCommon/c2f79c22e8678241b030c71523eb300c.

[58] CDE of NMPA. Technical Guideline for Clinical Research and Development of Rare Disease Drugs (Trial). https://www.cde.org.cn/main/news/viewInfoCommon/c4e1ef312a0a0c039a7a4ca55b91d4e8.

[59] CDE of NMPA. Technical Guideline for Clinical Research and Development of Rare Disease Drugs (Trial). https://www.cde.org.cn/main/news/viewInfoCommon/058e0d665b785e79b7f4f24dc1dc970c.

[60] CDE of NMPA.. Guideline for Natural History Studies in Drug Development for Rare Diseases (Draft for Comment). https://www.cde.org.cn/main/news/viewInfoCommon/74f7b4d7787d8dd5d2887e207e53d55c.

[61] CDE of NMPA. Technical Guideline for the Suitability of Single-arm Clinical Trials to Support the Marketing Application of Antineoplastic Drugs (Draft for Comment). https://www.cde.org.cn/main/news/viewInfoCommon/81bf3b56b627d7037ac849d0061e583c.

[62] CDE of NMPA. Clinical Research and Development of New Drugs for Traditional Chinese Medicine Compound Preparations Based on Experience (Trial). https://www.cde.org.cn/main/news/viewInfoCommon/8a1682a8d37494732f7f441dd11f5af6.

[63] CDE of NMPA. Technical Guideline for Communication under Registration and Review Evidence System Based on "Three Combinations" (Trial). https://www.cde.org.cn/main/news/viewInfoCommon/8a1682a8d37494732f7f441dd11f5af6.

[64] National Health Commission of the People’s Republic in China. Guideline for the Management of Comprehensive Clinical Evaluation of Pharmaceutical Products (Trial 2021 edition). http://www.nhc.gov.cn/yaozs/s2908/202107/532e20800a47415d84adf3797b0f4869.shtml.

[65] National Centre for the Comprehensive Assessment of Drugs and Health Technology. Technical Guideline for Comprehensive Clinical Evaluation of Pediatric Drugs (Trial edition 2022). http://www.nhei.cn/nhei/znfb/202206/c01d87a290664b01bf42a9dad769d69f/files/60c39855fa6048e6aae17323b1fbd546.pdf.

[66] National Centre for the Comprehensive Assessment of Drugs and Health Technology. Technical Guideline for Comprehensive Clinical Evaluation of Cardiovascular Drugs (Trial edition 2022). http://cpia.net.cn/uploads/20220630/59831d29ddfe35745066ea54956a9b0f.pdf.

[67] National Centre for the Comprehensive Assessment of Drugs and Health Technology. Technical Guideline for Comprehensive Clinical Evaluation of antitumor Drugs (Trial edition 2022). http://www.nhei.cn/nhei/znfb/202206/c01d87a290664b01bf42a9dad769d69f/files/4e062c199b17474ca680da5aac3b6d89.pdf.

[68] Tang N, Wang Z (2016). Comparison of bevacizumab plus chemotherapy with chemotherapy alone in advanced non-small-lung cancer patients. Onco Targets Ther.

[69] Xing P, Mu Y, Wang Y (2018). Real world study of regimen containing bevacizumab as first-line therapy in Chinese patients with advanced non-small cell lung cancer. Thorac Cancer.

[70] Li XY, Wang L, Wen Y (2018). Comparative effectiveness study of first-line pemetrexed-platinum doublet chemotherapy with or without bevacizumab for Chinese patients with advanced non-squamous non-small-cell lung cancer. J Clin Oncol.

[71] CDE of NMPA. Annex 1: JXHS2000131_Pratinib Capsules_Public Announcement of Marketed Drugs. https://www.cde.org.cn/main/xxgk/postmarketpage?acceptidCODE=6382d5c33c510a8b754b9471fac48d85.

[72] Li A, Liu B, Zong XY (2021). Research and development strategy of ancient famous Chinese Medicine and Practice analysis of Qingfei Detoxu Granules on the market. Zhong Yi Za Zhi.

[73] Pang WT, Yang FW, Zheng WK (2022). Clinical efficacy evaluation of Xuanfei Xuedu Granules in treating novel coronavirus pneumonia infected by Omicron strain. Tianjin J Tradit Chin Med.

[74] CDE of NMPA. JXHS2101001_Pralsetinib Capsules_Public Announcement of Marketed Drugs. https://www.cde.org.cn/main/xxgk/postmarketpage?acceptidCODE=55d73dc172b27aefcccf8aaec07abadd.

[75] Behind China's First New Drug to Market Based Entirely on Real-World Data: Commercialisation is a Year and a Half Faster, at Less Than a Quarter of the Cost of Bridging Trials. http://www.eeo.com.cn/2021/0417/485232.shtml.

[76] CDE of NMPA. JXHS2100035_Fluocinolone Acetonide Intravitreal Implants_Public Announcement of Marketed Drugs. https://www.cde.org.cn/main/xxgk/postmarketpage?acceptidCODE=5e189ea56f9c4d1fefe3d37de7d437f4.

[77] CDE of NMPA. JXHS2101087_Trilaciclib_Public Announcement of Marketed Drugs. https://www.cde.org.cn/main/xxgk/postmarketpage?acceptidCODE=2ce9bae53de6b90a3561ad317db27040.

[78] Evaluation of TRILACICLIB in Chinese Patients With Extensive-stage Small Cell Lung Cancer (ES-SCLC) for Chemotherapy-induced Myelosuppression, Antitumor Effects of Combination Regimens, and Safety in a Real-world Study. https://clinicaltrials.gov/ct2/show/NCT05071703.

[79] U.S. FOOD & DRUG ADMINISTRATION. Sentinel System Five-Year Strategy: 2019–2023. https://www.fda.gov/safety/fdas-sentinel-initiative/fdas-sentinel-initiative-background.

